# Qualitative study investigating the commissioning process for older people's services provided by third sector organisations: SOPRANO study protocol

**DOI:** 10.1136/bmjopen-2015-010724

**Published:** 2016-05-18

**Authors:** Gina Sands, Neil Chadborn, Chris Craig, John Gladman

**Affiliations:** 1 NIHR Collaboration for Leadership in Applied Health Research and Care East Midlands (CLAHRC EM), Institute of Mental Health, University of Nottingham, UK; 2 Division of Rehabilitation and Ageing, School of Medicine, University of Nottingham, UK

**Keywords:** Older adults, Third sector, Voluntary, Service Provision, CVS, Commissioning

## Abstract

**Introduction:**

The commissioning of third sector services for older people may influence the quality, availability and coordination of services for older people. The SOPRANO study aims to understand the relationships between and processes of commissioning bodies and third sector organisations providing health and social care services for older people.

**Methods and analysis:**

This qualitative study will be based in the East Midlands region of England. An initial scoping survey of commissioners will give an overview of services to maintain the health and well-being of older people in the community that are commissioned. Following this, semistructured interviews will be conducted with 4 sample groups: health and social care commissioners, service provider managers, service provider case workers and older service users. A sample size of 10–15 participants in each of the 4 groups is expected to be sufficient to reach data saturation, resulting in a final expected sample size of 40–60 participants. Informed consent will be gained from all participants, and those unable to provide informed consent will be excluded. The interview data will be analysed by 2 researchers using framework content analysis.

**Ethics and dissemination:**

Approval for the study has been gained from the University of Nottingham School of Medicine ethical review board, and the relevant approvals have been gained from the National Health Service (NHS) research and development departments for interviewing NHS staff. Early engagement with a wide range of stakeholders will ensure that the research findings are extensively disseminated to relevant stakeholders (including commissioners and third sector providers) in an accessible format using the extensive communication networks available to the National Institute for Health Research (NIHR) Collaboration for Leadership in Applied Health Research and Care CLAHRCs (applied health research organisations covering all of England). The study will also be disseminated through academic routes such as conference presentations and journal papers.

**Strengths and limitations of this study**

- This study will provide new information about the commissioning process and how this influences third sector services for older people. This will enable better exchanges between commissioning bodies and third sector organisations which may enhance the experience and care of older people.
- The study will incorporate the views of older service users which will bring a new perspective on commissioned health and social care services.
- As an applied research study, dissemination and implementation are important aspects of the project; therefore, this will give new insights on how best to connect with stakeholders.
- A limitation of this study would be that it is based in one geographical region (the East Midlands of England) and therefore may not be representative of commissioning systems in other areas.

## Introduction

Preventing ill health and remaining independent is a priority for many older people. The concept of ‘resilience’ as applied to older people could provide a framework to achieve this priority. Resilience is defined as ‘the process of effectively negotiating, adapting to, or managing significant sources of stress or trauma’, using assets and resources to bounce back in the face of adversity.^1^ Resilience is multifaceted and includes psychological, mobility, financial, environmental, physical, social and cultural factors,^2^ all of which are potentially open to multiple, low-level, interventions such as groups to address loneliness, household help and financial advice.

Many of the low-level health and social care services for older people are delivered by the third sector through charities and voluntary organisations. However, in the UK, these services are often commissioned through public bodies such as local authorities and the National Health Service (NHS). Commissioning in health and social care refers to the process of assessing needs, setting priorities, allocating resources (including minimising cost and risk), influencing providers and involving patient or public representatives.^3^ Commissioning also needs to ensure high quality and value for money care which meets patient needs.^4^ Health commissioning has changed considerably over the past two decades, with different organisational structures including health authorities, primary care groups, primary care trusts and now clinical commissioning groups (CCGs).^5^ There has been a recent drive towards greater integration of health and social care, with joint commissioning being one of the policy responses to this concern.^3^ There is also evidence of increasing complexity and uncertainty in social care commissioning, with further issues arising from integrating with healthcare commissioning processes.^6^ A prior history of collaboration between local authorities and health commissioners has been cited as one of the facilitators of successful joint commissioning, along with good communication and alignment of internal processes.^3^

**Figure 1:**
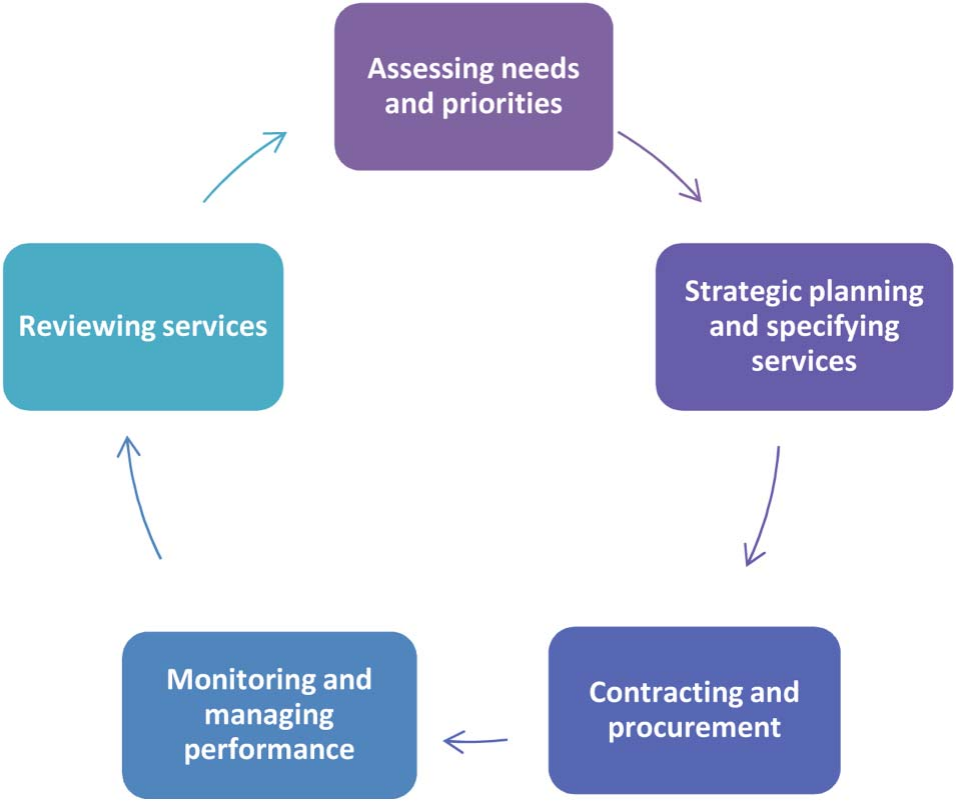
Commissioning cycle (adapted from Shaw *et al*^4^).

The commissioning process varies and no one way of commissioning has been favoured by the evidence; however, the basic commissioning cycle of needs assessment, priority setting, service development, procurement and review is well established.^7^ Therefore, the UK Department of Health have traditionally encouraged commissioners in the NHS to follow the general steps of assessing, planning, contracting, monitoring and reviewing as shown in figure 1.^4^

The funding models available through commissioning have also changed over recent years causing some concern in the third sector. The more traditional funding of block contracts and grants may be preferred by charities, with 75% stating that these funding types have a positive impact of the people they serve.^8^ More modern conditional contracts such as payment by results or tariff-based contracts received a less positive response from charities.^8^ Research in recent years has also questioned whether many third sector organisations are ‘commissioning ready’ and have the necessary skills and experience needed to win contracts for services.^9^

These issues demonstrate some of the potential challenges for commissioning third sector services for older people. With such complex commissioning practices and funding types, some third sector organisations may have difficulty navigating through the process, and therefore this could limit the amount of services available to older people. There also may be differences between the commissioning processes of NHS bodies and local authorities which could have an impact on the integration of health and social care services for older people. The aim of the SOPRANO study is to better understand the processes and relationships of commissioning third sector services for older people to help enable better interactions between the various stakeholders including commissioners, third sector providers and older people. This paper presents the protocol for the SOPRANO (Supporting Older People's Resilience through Assessing Needs and Outcomes) study using the SPIRIT checklist for standard protocol items^10^ and COREQ qualitative reporting criteria^11^ as a guide for content. The SOPRANO study will run from January 2014 to December 2016 and is currently in the recruitment phase.

## Methods and analysis

### Study setting and design

The study will be based within the East Midlands geographic region of the UK. The research will include participants from the NHS and local authority commissioning sector, the third sector (including voluntary organisations, charities and social enterprises), and older people using services in the community.

The SOPRANO project will employ a qualitative study design to understand the complex relationships and processes involved with commissioning third sector health and social care services for older people. This will involve a combination of a scoping survey of commissioners in the East Midlands region of the UK, and a series of interviews with stakeholders. The survey of commissioners will gather mainly qualitative data around the services and activities that have been commissioned for older people in the East Midlands. This will include the name of the service provider, what activities are undertaken, who the service is aimed at and how it came to be commissioned. The survey will be conducted electronically using Bristol Online Surveys (BOS), with access provided through targeted emails to commissioners.

The interviews will be undertaken with the following groups in the East Midlands: commissioners for health and social care; third sector managers or decision makers; third sector volunteers or case workers; and older service users. Interviews will be semistructured using an interview guide developed through consultation with the SOPRANO project external and public advisory group. This interview guide will be piloted with the first few participants in each subgroup and then reviewed by the research team and advisory group to ensure that it is fit for purpose and collecting useful data. Interviews will be carried out by two researchers with experience of interviewing research participants, and each interview is expected to last 45–60 min. The interviews will be conducted at a time and place convenient to the participant, such as university meeting rooms, community centres, participants' offices or another suitable quiet place. If the participant would prefer to be interviewed over the telephone, this will also be accommodated. Interviews will be audio recorded providing that this is acceptable to participants, interview notes will also be taken in case of equipment failure or participants who are unwilling to be recorded. These audio recordings will then be transcribed verbatim and anonymised to allow for analysis.

### Recruitment and sampling

A purposive sampling method will be employed for the survey of commissioners to ensure good representation of commissioners and services across the region. This will consist of targeted emails to commissioners in the East Midlands region, including CCGs (clinically led local statutory bodies), health and well-being boards (forums for key leaders from the health and care system to collaborate to improve health and well-being), public health departments, and adult social care departments (responsible for supporting people in the community). Following this initial contact, it is expected that sampling may snowball to include other commissioners that respondents may suggest as being suitable participants for the survey.

**Figure 2:**
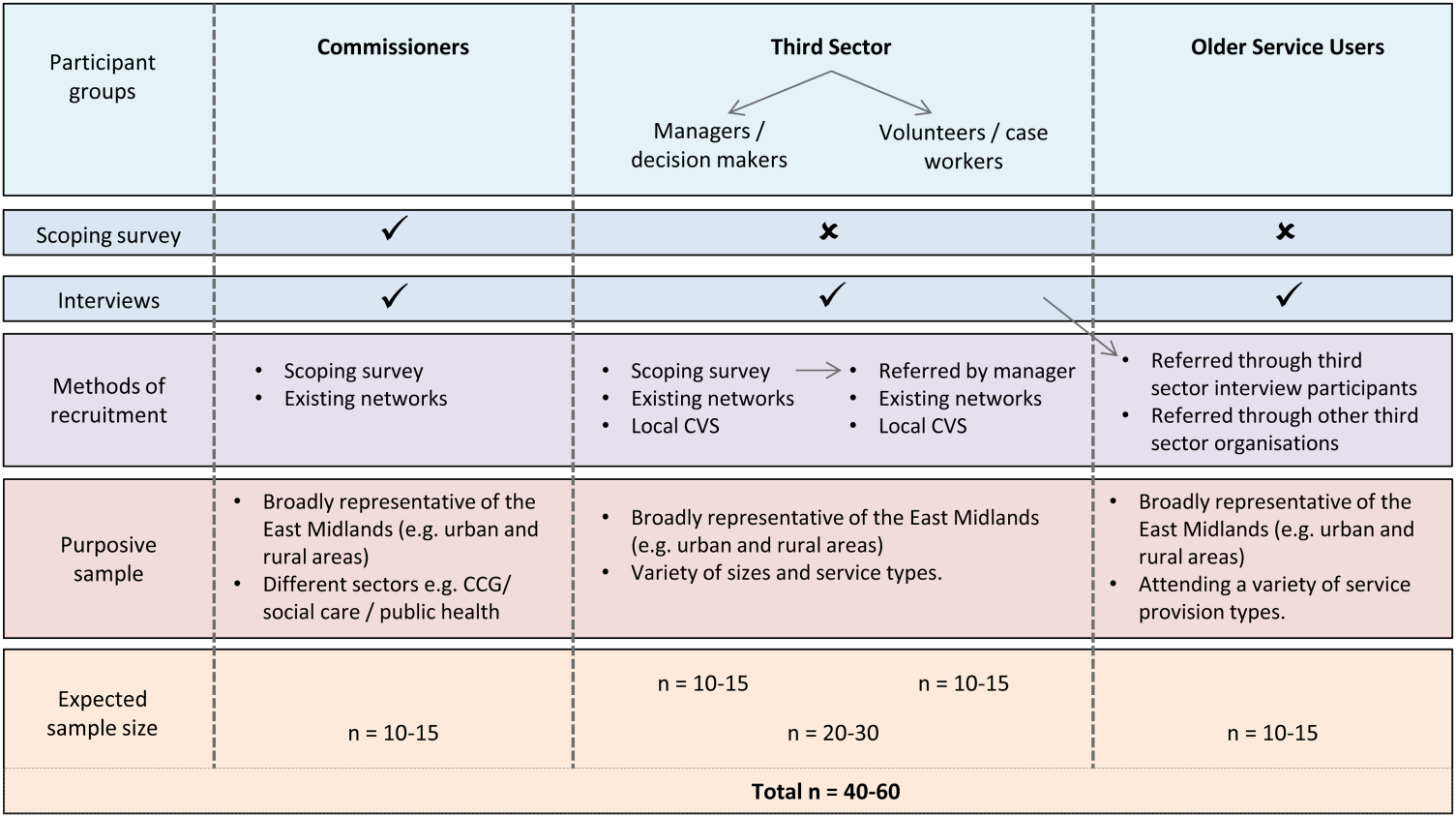
Study recruitment pathway. CCG, clinical commissioning group; CVS, Community and Voluntary Service.

The interview phase of the study will also be purposive using the results of the survey and networking around the East Midlands region to identify services that can be used as interesting case studies. These case studies may also be combined with other ‘stand-alone’ interviews of people across the region to ensure a comprehensive overview is offered. Four main groups of people will be sampled for the interview stage of the study: commissioners, service provider managers, service provider keyworkers/front-line staff and older people. The study will aim to recruit ∼10–15 participants in each group giving a total expected sample size of 40–60 participants, although the interviews will not continue if data saturation is reached before then. Potential participants will be selected for interview based on the locality to ensure there is a spread of rural and urban areas, and by different service types. Commissioners and service provider potential participants will be approached by the research team via email, post or in person. Older people will be first approached by the service providers/third sector organisations they attend who will act as gatekeepers. The service provider will ask if they are happy to receive information, and if yes then the research team will make contact. Informed consent will be gained from all participants. Participants will be excluded if they are unable to give informed consent or if data saturation is reached. A diagram of the study recruitment pathway is shown in figure 2.

### Analysis

The scoping survey of commissioners will be summarised using content analysis to understand the variety and geographical range of services across the East Midlands. These survey findings will then inform the design and sampling of the main interview phase of the study.

Interview data from audio recordings will be transcribed verbatim prior to analysis. The analysis of interview data will be conducted using a qualitative framework approach with the coding framework derived from the themes of questioning and additional codes emerging from the data. The analysis will be shared by two researchers experienced in qualitative interview analysis, each leading the coding on approximately half of the sample and then checking coding on the remaining half. If new codes emerge from the data, then the whole sample will be rechecked to ensure consistency of coding. The data management and analysis will be aided by using the qualitative data analysis software QSR NVivo V.10. Following data coding, themes will be generated independently by the two researchers and then discussed until consensus is reached. Themes from each subgroup of participants will then be triangulated to explore any commonalities or differences between groups. The data from older people in particular are expected to provide a different perspective on the issues of needs assessment, outcomes and coordinated care that may be provided by the other profession-based sample groups. Emergent findings will also be discussed within the wider SOPRANO research team and the study's external and public advisory group to gain different perspectives on the data.

## Ethics and dissemination

### Ethics and oversight

The project also has governance approval from the relevant research and development departments for the survey and interviews with NHS commissioners which were deemed to be a service evaluation for this purpose. Informed consent will be gained from each interview participant before the interview begins; informed consent to the survey will be indicated by a check box on the online survey form. If participants require any special provision for informed consent, all endeavours will be made to provide the required format (eg, large print, braille, language interpreters).

The SOPRANO project also meets regularly with the relevant East Midlands Collaboration for Leadership in Applied Health Research and Care (CLAHRC) research theme which provides oversight and guidance, along with regular reporting to CLAHRC East Midlands management and the National Institute for Health Research (NIHR). There is also an external and public advisory group consisting of members of the public, third sector groups, health professionals, commissioners and academics which provides additional oversight and guidance to the project.

Data will be kept securely and confidentially. All hard copy data will be kept in a locked cabinet inside an access controlled room at the University of Nottingham; electronic data will be password protected and accessible only to the research team.

### Dissemination

The SOPRANO study has a dissemination plan to ensure the research is widely distributed and has impact both within and outside academia. The findings will be published in academic journals and relevant conferences towards the end of the project in 2016. These papers will also be converted into CLAHRC Brokering Innovation Through Evidence (BITEs) which are short half page summaries aimed to be accessible to commissioners and other professional stakeholders.^12^ Alongside this, links have been made from the beginning of the study with various stakeholders in the third sector, commissioning bodies and public groups to ensure that the implementation of research is a continual process throughout the study. The research team will also engage with stakeholders of ageing research (including older people) through the East Midlands Research into Ageing Network (EMRAN). Participants will not be identified in any publications or dissemination activities; confidentiality will be ensured by using study codes to differentiate participant quotations.
